# Near-real-time daily estimates of fossil fuel CO_2_ emissions from major high-emission cities in China

**DOI:** 10.1038/s41597-022-01796-3

**Published:** 2022-11-10

**Authors:** Da Huo, Kai Liu, Jianwu Liu, Yingjian Huang, Taochun Sun, Yun Sun, Caomingzhe Si, Jinjie Liu, Xiaoting Huang, Jian Qiu, Haijin Wang, Duo Cui, Biqing Zhu, Zhu Deng, Piyu Ke, Yuli Shan, Olivier Boucher, Grégoire Dannet, Gaoqi Liang, Junhua Zhao, Lei Chen, Qian Zhang, Philippe Ciais, Wenwen Zhou, Zhu Liu

**Affiliations:** 1 Department of Earth System Science, Tsinghua University, Beijing, 100084 China; 2 Department of Civil & Mineral Engineering, University of Toronto, Toronto, ON M5S 1A1 Canada; 3 Product and Solution and Website Business Unit, Alibaba Cloud, Hangzhou, Zhejiang, 311121 China; 4 School of Environmental Science and Engineering, Tianjin University, Tianjin, 300072 China; 5 Department of Electrical Engineering, Tsinghua University, Beijing, 100084 China; 6 The Chinese University of Hongkong, Shenzhen, Guangdong, 518172 China; 7 The Shenzhen Institute of Artificial Intelligence and Robotics for Society, Shenzhen, Guangdong 518172 China; 8 School of Geography, Earth and Environmental Sciences, University of Birmingham, Birmingham, B15 2TT UK; 9 Institute Pierre-Simon Laplace, Sorbonne Université/CNRS, Paris, France; 10 Robert M. Buchan Department of Mining, Queen’s University, Kingston, ON K7L 3N6 Canada; 11 Laboratoire des Sciences du Climate et de l’Environnement LSCE, Orme de Merisiers, 91191 Gif-sur-Yvette, France

**Keywords:** Climate-change adaptation, Energy modelling, Civil engineering

## Abstract

Cities in China are on the frontline of low-carbon transition which requires monitoring city-level emissions with low-latency to support timely climate actions. Most existing CO_2_ emission inventories lag reality by more than one year and only provide annual totals. To improve the timeliness and temporal resolution of city-level emission inventories, we present Carbon Monitor Cities-China (CMCC), a near-real-time dataset of daily CO_2_ emissions from fossil fuel and cement production for 48 major high-emission cities in China. This dataset provides territory-based emission estimates from 2020-01-01 to 2021-12-31 for five sectors: power generation, residential (buildings and services), industry, ground transportation, and aviation. CMCC is developed based on an innovative framework that integrates bottom-up inventory construction and daily emission estimates from sectoral activities and models. Annual emissions show reasonable agreement with other datasets, and uncertainty ranges are estimated for each city and sector. CMCC provides valuable daily emission estimates that enable low-latency mitigation monitoring for cities in China.

## Abstract (summary)

**Table:** 

Measurement(s)	carbon dioxide emissions
Technology Type(s)	fossil fuel consumption

## Background & Summary

City-level greenhouse gas (GHG) emissions data is crucial for monitoring and planning urban climate change mitigation efforts^1–4^. Recent study has estimated that the top 25 cities worldwide accounted for over 50% of the total urban GHG emissions^5^, which highlights the importance of targeting major cities for achieving carbon neutrality goals. As the largest emitter and developing country in the world, China has committed to reach carbon peak by 2030 and carbon neutrality by 2060. Currently, carbon dioxide (CO_2_) emissions from cities in China account for more than 80% of its national scope-1 and scope-2 fossil-fuel CO_2_ emissions^6^, which put the fast-expanding Chinese cities at the frontline of their ambitious climate targets. Many cities have set their own emission mitigation targets, which require them to monitor and report emissions on a timely basis^5^. Unfortunately, most existing CO_2_ emission inventories are either calculated at a national/provincial scale or downscaled from national inventories using proxies that encompass large uncertainties at city level^7,8^.

City-level CO_2_ emission refers to the CO_2_ emission generated within the a city’s territory and the emissions associated with the a city’s consumption which may occur outside the city’s territory^9^. Based on the origin of emissions, the in-boundary emissions are typically classified as scope-1, emissions associated with imported electricity are typically classified as scope-2, and scope-3 refers to emissions generated from other trans-boundary activities. In this study, we estimate scope-1 emissions for cities. Many existing CO_2_ emission datasets for China are constructed at national or provincial level with a time lag of 2 + years, such as the China Emission Accounts and Datasets (CEADs: https://www.ceads.net/) and the Multi-resolution Emission Inventory (MEIC, http://meicmodel.org/). Furthermore, very few cities disclose their emissions, and the coverage and reliability of inventories based on self-reported data from city governments and similar organizations are difficult to assess^3,10^. China High-Resolution Emission Database (CHRED) and China City Carbon Dioxide Emissions Dataset^11^ are the only recent city-level emission inventories for China, but their city-level updates are released every five 5 years with a temporal resolution of one year, which can not meet the soaring demand for near-real-time (NRT) high temporal resolution emission inventories.

City-level emission datasets provide key information for identifying emissions trends across different communities, social classes and functional urban areas, which helps generate a deeper understanding of the relationships between urbanization and emissions, especially for developing countries, which in turn can inform urban climate change mitigation actions. The fifth Intergovernmental Panel on Climate Change (IPCC) report^12^ has also highlighted the urgent need for comprehensive city-level CO_2_ emission inventories. Given the limited timeliness and quality in existing inventories, most cities have strong demands for low-latency CO_2_ emission datasets for more effective and easily-traceable urban climate actions^1,5,13–16^. The future of global decarbonization may rely more and more on the rapidly expanding cities like those in China, where timely emission datasets are lacking. Monitoring city-level CO_2_ emissions at a higher temporal resolution will provide us with critical information to tackle the climate change crisis by facilitating local governments in policymaking and mitigation efficacy assessment^17–22^. Recent studies have successfully estimated NRT daily CO_2_ emissions for investigating the impacts of COVID-19 on global CO_2_ emissions^17,19,23^. Here we show that an improved framework can be applied at city scale.

The 48 major cities in this dataset are selected to represent the landscape of the emission from Chinese cities. Specifically, we select cities based on four criteria: 1. direct-administered municipalities, which are mostly mega-cities (e.g., Beijing), 2. provincial capitals (e.g., Guangzhou), 3. high-emission industrial cities (e.g., Tangshan), and 4. high-GDP cities (e.g., Suzhou). all provincial capitals (typically the largest city in a province) are covered to represent cities in each geographic regions, other high-emission and high-GDP cities can reasonably represent the major industrial and commercial activities. We estimated that the total emissions from these 48 cities combined account for about 43.8% of the total emissions of entire China in 2020 (4.6 *GT* over 10.5 *GT* CO_2_ based on Carbon Monitor^19,24^, excluding shipping), while only hold 21% of the total population. This also demonstrates that in addition to the overall national mitigation targets, which can be too general or impractical for individual cities, zooming in to key cities with localized actions could be more effective.

Here we present the methodology for producing Carbon Monitor Cities-China (CMCC), a near-real-time daily fossil fuel CO_2_ emission dataset for 48 major high-emission cities in China. This dataset provides scope-1 emission estimates from 2020 to 2021 for five sectors: power generation, residential (buildings and services), industry, ground transportation, and aviation. Innovative methods are developed and latest city-specific fossil fuel consumption data are used to integrate bottom-up annual inventories with daily emission estimates from sectoral activities and models. We hope this dataset can provide cities and decision-makers with timely information critical to assessing the efficacy of mitigation policies. This dataset can also support the scientific community for better understanding city-level daily emission dynamics.

## Methods

### Workflow Overview

**Fig. 1:**
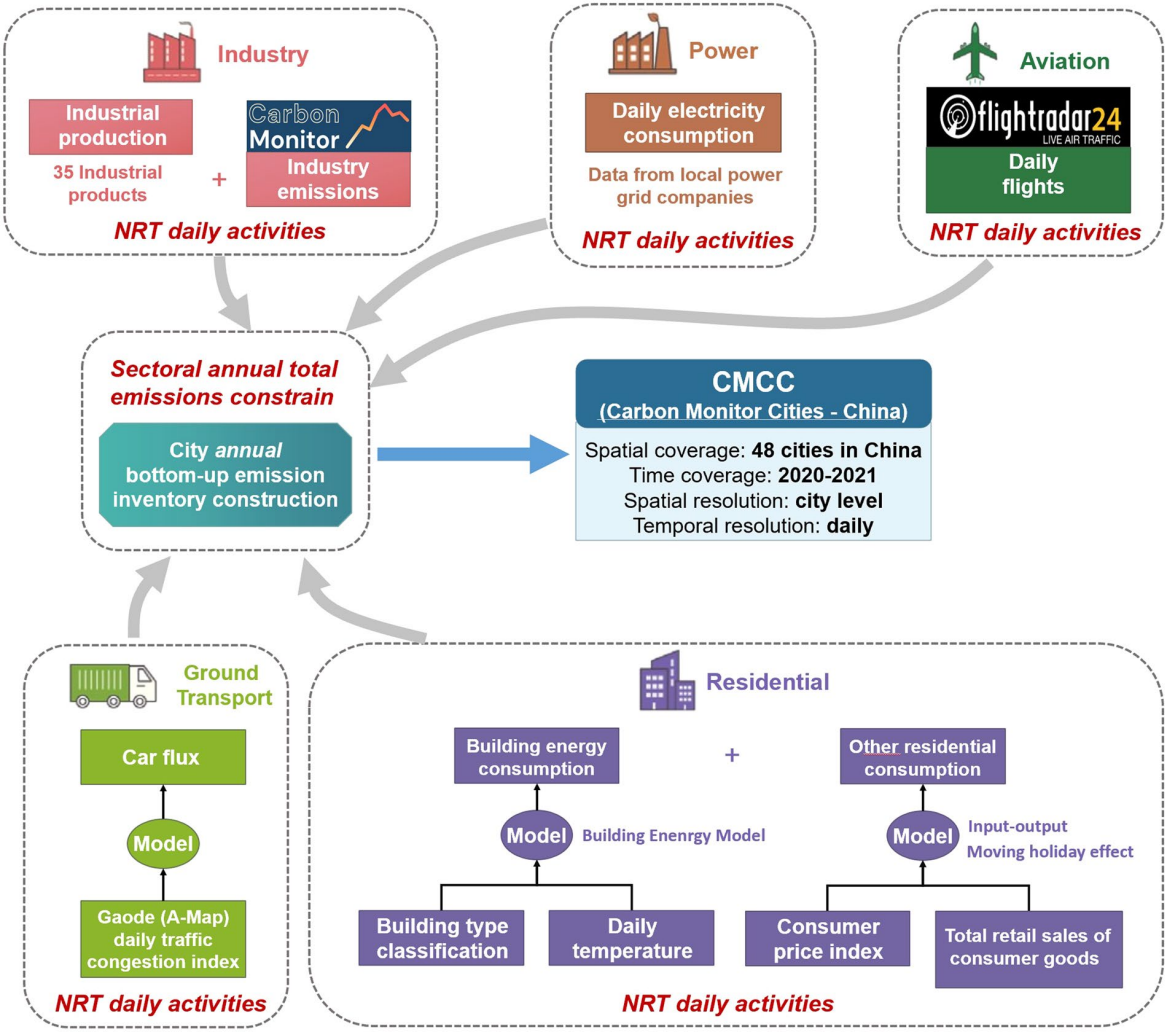
Flowchart illustrates the main workflow, data, and model used in each sector.

CMCC is constructed following a three-stage workflow (Fig. 1).

- The first stage is the construction of annual emission inventories for each city using bottom-up fossil fuel consumption data following IPCC administrative territorial-based accounting methods.
- In the second stage, we collect daily activity data for each sector and develop related models to estimate daily variation in emissions for each city.
- The final step is to disaggregate annual emissions to daily scale using results from the second stage, so that the annual sectoral total emissions are consistent with results from the first stage.

For quality assurance, we collected city-level inventories from other datasets (which only provide annual results) and compared them to our results for validation. We then performed uncertainty analysis to estimate the uncertainty ranges for each sector accounting for both uncertainties from the annual bottom-up inventories and daily activities. The detailed procedures for these steps are described in later sections. The construction of this dataset follows a different workflow as compared to the global Carbon Monitor Cities dataset we have recently published^20^. The global dataset is generated from a top-down spatial downscaling workflow, which does not incorporate energy statistics/inventory data for individual cities. In this work, daily emission estimates are constrained by bottom-up inventories collected from cities, therefore, although the coverage of this dataset is smaller, the accuracy is improved.

**Fig. 2:**
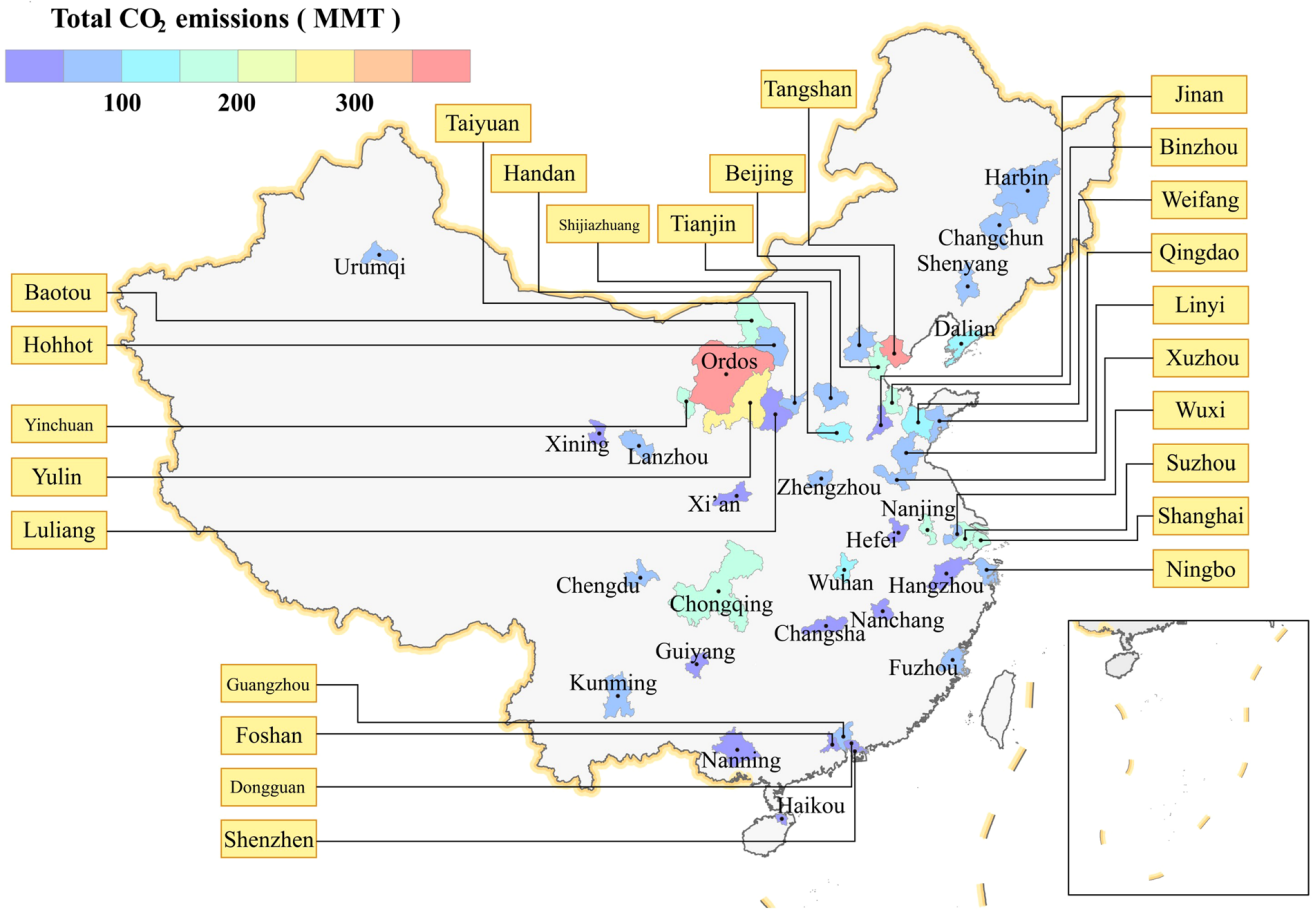
Map showing the administrative areas of all 48 cities covered in this dataset and their total emissions in year 2020.

CMCC covers 48 cities in China and their administrative areas are shown in Fig. 2. These cities were selected based on four criteria: 1. direct-administered municipalities, 2. provincial capitals, 3. high-emission industrial cities, and 4. high-GDP cities. Table 1 lists all 48 cities and the classifications. Currently, CMCC provides city-level emission inventories from 01/01/2020 to 31/12/2021 for five main sectors: 1. Power generation, 2. Residential (buildings and services), 3. Industrial production, 4. Ground transportation, and 5. Aviation.

**Table 1:** List of the 48 cities with classifications.

City	Province	Classification	City	Province	Classification
Beijing	Beijing	case 1, municipality	Wuxi	Jiangsu	case 3, high-GDP
Tianjin	Tianjin	case 1, municipality	Hangzhou	Zhejiang	case 3, provincial capital
Chongqing	Chongqing	case 1, municipality	Zhengzhou	Henan	case 3, provincial capital
Tangshan	Hebei	case 1, high-emission	Fuzhou	Fujian	case 3, provincial capital
Shanghai	Shanghai	case 1-1, municipality	Binzhou	Shandong	case 3, high-emission
Qingdao	Shandong	case 2, high-GDP	Haikou	Hainan	case 3, provincial capital
Changsha	Hunan	case 2, provincial capital	Guiyang	Guizhou	case 3, provincial capital
Nanjing	Jiangsu	case 2, provincial capital	Jinan	Shandong	case 3, provincial capital
Baotou	Inner Mongolia	case 2, high-emission	Nanchang	Jiangxi	case 3, provincial capital
Taiyuan	Shanxi	case 2, provincial capital	Xi’an	Shaanxi	case 3, provincial capital
Dalian	Liaoning	case 2, high-GDP	Yinchuan	Ningxia	case 3, provincial capital
Dongguan	Guangdong	case 2, high-GDP	Nanning	Guangxi	case 3, provincial capital
Foshan	Guangdong	case 2, high-GDP	Hohhot	Inner Mongolia	case 3, provincial capital
Ningbo	Zhejiang	case 2, high-GDP	Chengdu	Sichuan	case 3, provincial capital
Handan	Hebei	case 2, high-emission	Shenyang	Liaoning	case 3, provincial capital
Xuzhou	Jiangsu	case 2-1, high-emission	Hefei	Anhui	case 3, provincial capital
Yulin	Shaanxi	case 2-1, high-emission	Harbin	Heilongjiang	case 3, provincial capital
Weifang	Shandong	case 2-1, high-emission	Linyi	Shandong	case 3-1, high-emission
Xining	Qinghai	case 2-1, provincial capital	Urumqi	Xinjiang	case 3-1, provincial capital
Changchun	Jilin	case 2-1, provincial capital	Lanzhou	Gansu	case 3-1, provincial capital
Shijiazhuang	Hebei	case 2-1, provincial capital	Luliang	Shanxi	case 3-1, high-emission
Shenzhen	Guangdong	case 3, high-GDP	Kunming	Yunnan	case 3-1, provincial capital
Guangzhou	Guangdong	case 3, provincial capital	Wuhan	Hubei	case 3-1, provincial capital
Suzhou	Jiangsu	case 3, high-GDP	Ordos	Inner Mongolia	case 3-1, high-emission

### City-Level Annual CO_2_ Emissions Inventory Construction

According to the IPCC guidelines, city-level CO_2_ emissions can be calculated by multiplying the fossil fuel consumption data (*FC* for sector *s* and fuel *f*) and the corresponding emissions factors (*EF*) (Eq. 1)^25^:

1 $$Emis=\sum \sum F{C}_{s,f}\times E{F}_{s,f}.$$

Annual CO_2_ emission inventories consist of two parts, fuel-related emissions and process-related emissions. For fuel-related activity data, Energy Balance Table (EBT), Energy Processing and Conversion Table (EPCT), Industry Sectoral Energy Consumption (ISEC) are used to calculate fuel-related emissions, while cement production is covered in the process-related emissions. An EBT is an aggregate of energy production, transformation, and final consumption. EBTs for cities are released by the National Bureau of Statistics of provincial and municipal governments in China. Sectoral consumption of fossil fuels from EBT is used as fossil fuel consumption data to calculate fuel-related emissions. EPCT presents energy input and output during transformation process to complement EBT for cities without city-level EBT. Industry is responsible for a large portion of city CO_2_ emissions in China^26^, so we use ISEC to calculate industrial emissions separately based on energy activity data for industrial sub-sectors^27^. EBTs, EPCTs and ISECs are acquired from China Energy Statistical Yearbook and city-specific statistical yearbooks. Annual fossil fuel consumption data are acquired from China Energy Statistical Yearbook and city-specific statistical yearbooks, which are the most commonly used data sources for constructing emission inventories for China^28^. Since the 2021 China Energy Statistical Yearbook and the 2021 statistical yearbooks for some cities (Shijiazhuang and Handan) have not been released, we compute and use the relative changes in aggregated daily activity data (detailed in later sections) for 2020 versus 2021 and the Carbon Monitor dataset (https://carbonmonitor.org)^17,29,30^ to estimate NRT energy consumption in year 2021.

**Fig. 3:**
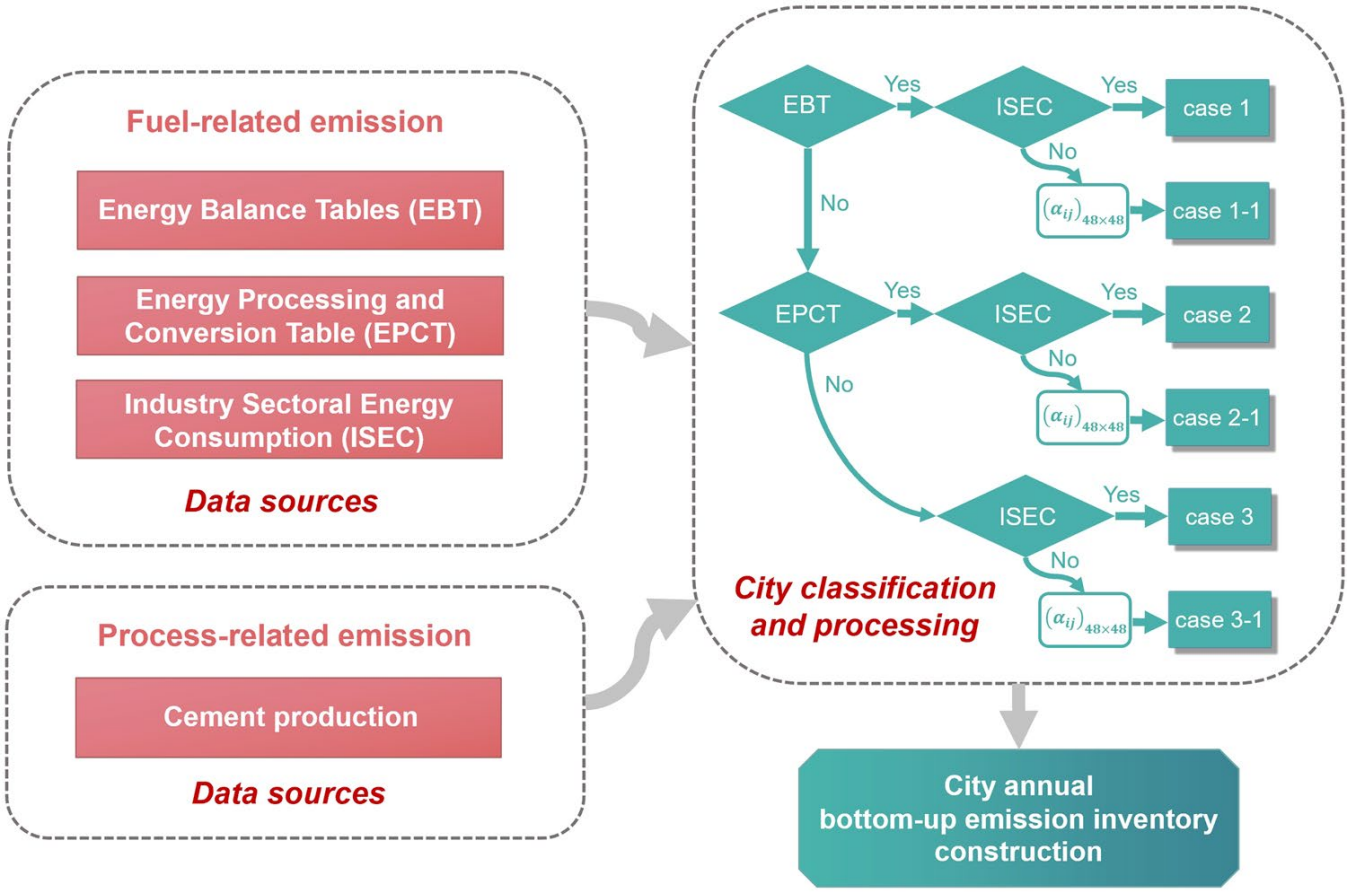
City annual CO_2_ emissions inventory construction framework for 48 cities. (*α*_*ij*_)_(48×48)_ refers to the similarity matrix of 48 cities for identifying a similar city to estimate missing ISEC.

For the selected 48 cities in China, emissions from 34 cities are calculated with above-mentioned energy statistics according to the approach and emission factors from Shan *et al*.^27^, in which cities are classified as case 1, case 2 and case 3 based on the degree of completeness of their fossil fuel consumption data, each case is processed differently. The specific city classification and processing is shown in Table 1 and Fig. 3.

For other 14 cities without ISEC, we introduce a city similarity matrix, which can be used to identify a similar city to each of the 14 cities to estimate the missing ISEC. The similarity matrix is constructed based on the socioeconomic indicators proposed by Jing *et al*.^31^ which governs city-level CO_2_ emissions, including GDP, population, industrial output, urbanization rate and GDP per capita. Due to the regional coordinated development in China, cities in the same region have similar industrial structures, such as Changsha and Wuhan, so we also take the geographic distance between two cities as one of the indicators for constructing the similarity matrix (Table 2). For each indicator, we compute the Euclidean distance between two cities, and apply a 0-1 normalization. The closer euclidean distance for all indicators between cities refers to the higher similarity, otherwise the lower similarity (Eqs. 2, 3). Based on this approach, we calculate the city similarity matrix for all 48 cities to cover the missing city-level ISEC energy consumption data. For example, to estimate the ISEC for Weifang, which is not available, we identify that Xuzhou is the most similar to Weifang, however, ISEC is also unavailable for Xuzhou, we can then select the city with the second highest similarity (0.92), Linyi as a proxy for estimating ISEC for Weifang based on Linyi’s sectoral partition coefficient (Eq. 4), note that only the share of each industry in ISEC rather than the total energy consumption is used for substitution. Also note that in this example, both cities are geographically close to each other, which highlights the impact of geographic distance on determining the similarity.

2 $$C={({\alpha }_{ij})}_{48\times 48},$$

3 $${\alpha }_{ij}=1-{\left[\mathop{\sum }\limits_{k=1}^{5}{\left({x}_{i,k}-{x}_{j,k}\right)}^{2}+{d}^{2}\left({l}_{i},{l}_{j}\right)\right]}^{1/2}/\sqrt{6},$$

where *C* is similarity matrix of 48 cities, *α*_*ij*_ is the similarity between city *i* and city *j*. *α*_*ij*_ is between 0 and 1, 0 means two cities are completely unrelated, and 1 means two cities are identical. *x*_*i,k*_ is *k* th indicator of city *i*, *l*_*i*_ is the geographic location of *l*_*i*_ and *d*(*l*_*i*_, *l*_*j*_) is geographic distance between city *i* and city *j*. The complete city similarity matrix for all the 48 cities is included in the data file.

**Table 2:** Indicators used to construct the city similarity matrix.

Indicators index	Indicators
X1	GDP
X2	Population
X3	Industrial output proportion
X4	Urbanization ratio
X5	GDP per capita
X6	Geographic location

Cities without ISEC also need a proxy by similarity matrix, and we use ISEC of the similar city as sectoral partition coefficient and assign total energy consumption to sectoral data:

4 $$ISEC={E}_{total}\times ISE{C}^{{\prime} }/{E}_{total}^{{\prime} },$$

where *ISEC* is industrial sectoral energy consumption, *E*_*total*_ is total industrial energy consumption and *ISEC*′ is industrial sectoral energy consumption of a similar city, ${E}_{total}^{{\prime} }$ is total industrial energy consumption of a similar city. In the workflow (Fig. 3), these cities with missing data are classified as case 1-1, case 2-1 and case 3-1, respectively, in order to distinguish them from cities with complete industrial sectoral energy consumption (see below). Therefore, we have classified 48 cities into 6 groups. Each case is then processed differently to acquire the annual bottom-up inventories. For the 48 cities, there are 4 case 1 cities, 10 case 2 cities, 20 case 3 cities, 1 case 1-1 city, 6 case 2-1 cities, and 7 case 3-1 cities. Specifically:

- Case 1 cities: EBTs are available for the city.
- Case 2 cities: EBTs are absent, but EPCTs are available for the city.
- Case 3 cities: Both EBTs and EPCTs are absent for the city.
- Case 1-1 cities: Meet the conditions of case 1 but without ISEC, using similarity matrix to estimate.
- Case 2-1 cities: Meet the conditions of case 2 but without ISEC, using similarity matrix to estimate.
- Case 3-1 cities: Meet the conditions of case 3 but without ISEC, using similarity matrix to estimate.

According to sectoral coverage and correspondence with IPCC (Table 3), the total annual emissions are allocated to five main sectors. Sector correspondence with CEADs dataset (https://www.ceads.net) is used. Transport sector is divided into three parts, ground transportation, aviation and ships. We allocate them according to the proportion of the transportation sector in the 2020 China City Carbon Dioxide Emission Dataset published by the China City Greenhouse Gas Working Group (https://www.cityghg.com/). The sectoral annual emissions from city emission inventories are then used to constrain the sum of daily emission estimates.

**Table 3:** Sectoral coverage and correspondence with CEADs EBT and IPCC.

CMCC Sector	Coverage (CEADs EBT subsectors)	Corresponding CHRED Sector	Corresponding IPCC Sector
Power	Production and Supply of Electric Power, Steam and Hot Water	No dedicated power sector (mostly covered in industry)	1A1a: Public electricity and heat production
Industry			
35 industrial products: **Iron and steel** 1. Crude steel production 2. Crude iron ore **Chemical and petrochemical** 3. Sulfuric acid 4. Caustic soda 5. Soda ash 6. Ethylene 7. Chemical fertilizer 8. Chemical pesticide 9. Primary plastic and synthetic rubber 10. Plastic products 11. Chemical fiber **Non-ferrous metals** 12. ten kinds of nonferrous metals 13. Refined copper 14. Electrolyzed aluminum 15. Aluminum **Non-metallic minerals** 16. Cement and clinker production 17. Plain glass 18. Phosphate ore **Transport equipment** 19. Civil steel ships 20. Automobiles 21. Rail vehicles **Machinery** 22. Metal smelting equipment 23. Cement equipment 24. Industrial boilers 25. Mobile communication base station equipment 26. Large tractor 27. Medium tractor 28. Small tractor **Food and tobacco** 29. Salt 30. Sugar 31. Beverage **Paper, pulp and printing** 32. machine-made paper and paperboards 33. Newsprint **Textile and leather** 34. Yarn 35. Cloth	Coal Mining and Dressing, Petroleum and Natural Gas Extraction, Ferrous Metals Mining and Dressing, Nonferrous Metals Mining and Dressing, Nonmetal Minerals Mining and Dressing, Other Minerals Mining and Dressing, Logging and Transport of Wood and Bamboo, Food Processing, Food Production, Beverage Production, Tobacco Processing, Textile Industry, Garments and Other Fiber Products, Leather, Furs, Down and Related Products, Timber Processing, Bamboo, Cane, Palm Fiber, Straw Products, Furniture Manufacturing, Papermaking and Paper Products, Printing and Record Medium Reproduction, Cultural, Educational and Sports Articles, Petroleum Processing and Coking, Raw Chemical Materials and Chemical Products, Medical and Pharmaceutical Products, Chemical Fiber, Rubber Products, Plastic Products, Nonmetal Mineral Products, Smelting and Pressing of Ferrous Metals, Smelting and Pressing of Nonferrous Metals, Metal Products, Ordinary Machinery, Equipment for Special Purposes, Transportation Equipment, Electric Equipment and Machinery, Electronic and Telecommunications Equipment, Instruments, Meters, Cultural and Office Machinery, Other Manufacturing Industry, Scrap and waste, Construction	Industry	1A2: Manufacturing Industries and Construction, 1A1bc: Other Energy Industries, 2A1: Cement production
Residential (Buildings and Services)	Wholesale, Retail Trade and Catering Services, Others, Urban, Rural	Household, Service	1A4: Residential and other sectors
Ground Transport	Transportation, Storage, Post and Telecommunication Services	Road, Railway	1A3b: Road transportation no resuspension 1A3c: Rail transportation 1A3d: Inland navigation 1A3e:Other transportation
Aviation	Transportation, Storage, Post and Telecommunication Services	Aviation	1A3a: Domestic aviation 1C1: International aviation

### Near-Real-Time Daily Activities by Sector

CMCC disaggregates the annual emissions into each day using daily activity data and models for each sector (Fig. 1). This section describes the NRT daily activity data and models for each sector.

#### Power

For each city, annual emissions from power generation *Emis*_*power,y*_) can be disaggregated into daily emissions *Emis*_*power,d*_ using daily power generation data *GElec*_*c,d*_ and the corresponding daily generation-related emission factors *GEF*_*power,c,d*_ (Eq. 5):

5 $$\begin{array}{lll}Emi{s}_{power,d} & = & Emi{s}_{power,y}\times \frac{Emi{s}_{power,d}}{\sum Emi{s}_{power,d}}\\  & = & Emi{s}_{power,y}\times \frac{GEle{c}_{c,d}\times GE{F}_{power,c,d}}{\sum \left(GEle{c}_{c,d}\times GE{F}_{power,c,d}\right)},\end{array}$$

where subscripts *power*, *y*, *d*, *c* represent the power sector, year, day and city, respectively. Note that this dataset only covers scope-1 power emissions, meaning that if a city does not have any power generation plants within its territory (solely rely on imported electricity), then no power emissions are accounted for. For cities lacking daily power generation data, city-specific power consumption data (collected from local grid companies) is used to represent the power generation, then the daily emissions from power generation are computed as:

6 $$Emi{s}_{power,d}=Emi{s}_{power,y}\times \left(\frac{Emi{s}_{c,d,co}}{\sum Emi{s}_{c,d,co}}\right),$$

where the subscript *co* represents power consumption. Daily emissions from power consumption *Emis*_*c,d,co*_ is calculated with daily power consumption data from varying sources for different cities, and the daily power consumption data collected from the local power grid companies (those within a city’s administrative area) are the preferred data for use. The daily emissions from power consumption *Emis*_*c,d,co*_ are calculated as follows:

7 $$Emi{s}_{c,d,co}=CEle{c}_{c,d}\times CE{F}_{power,c,d},$$

where *CElec*_*c,d*_ and *CEF*_*power,c,d*_ are the daily power consumption data and the corresponding power consumption-related emission factors of the city, respectively.

The daily power-related emission factor is crucial for power emissions estimation^32^. The daily power consumption-related emission factor for a city *CEF*_*power,c,d*_ are assumed to be approximate to that of its mother province *CEF*_*power,p,d*_, as available data only support provincial level emission factor estimation. The daily power emission factor of the corresponding province is estimated by the daily power generation mix with the average emission factor of different generation types and the net imported power with their emission factors following a recent study^33^:

8 $$CE{F}_{power,c,d}\approx CE{F}_{power,p,d}=\frac{{\sum }_{f}G{E}_{power,f,d}\times GE{F}_{power,f}+{\sum }_{op}I{E}_{power,op,d}\times IE{F}_{power,op}}{{\sum }_{f}G{E}_{power,f,d}+{\sum }_{op}I{E}_{power,op,d}},$$

where the subscript *f* represents the generation type, including coal-fired generation, gas-fired generation, oil-fired generation, and so on; *IE*_*power,op,d*_ and *IEF*_*power,op*_ represent the daily net power imported from other province *op* and the corresponding emission factor, respectively; *GEF*_*power,f*_ is the average simple operating margin CO_2_ emission factor of the generation type *f* in China. We assume that the emissions from total power generation are equal to the emissions from thermal power generation since the thermal power generation contributes over 90% of the emissions from total power generation.

For cities with complete daily power consumption data covering the whole accounting period (2020 to 2021 in this dataset), outliers are removed, and missing values are filled with interpolation. For cities with incomplete daily power consumption data, the daily consumption data are simulated based on annual power consumption data and provincial-level daily load curves which are also collected. Therefore, daily power consumption data *CElec*_*c,d*_ can be calculated with the daily load curve and the annual power consumption of the city.

Normally, the load curve of a city has highly similarity in adjacent years, and the load pattern of the previous year could represent that of the next few years. However, COVID-19 has led to China’s national lockdown in early 2020 and its impact should also be considered. The daily load pattern of a city is often similar to its mother province. Thus, the provincial daily load pattern is used to represent the cities in it. We first use the normalized daily load curve of provinces in 2019 to represent the daily load pattern of the corresponding cities from 2020 to 2021, then modify the power sector curves to reflect the impact of COVID-19 national lockdown in early 2020. The daily load curve in 2019 can be obtained from the local power grid companies. The annual power consumption of the cities can be acquired from the National Bureau of Statistics or China City Statistical Yearbook. The daily power consumption of the cities *CElec*_*c,d*_ can be calculated as follows:

9 $$CEle{c}_{c,d}=\frac{CEle{c}_{p,d}}{\sum CEle{c}_{p,d}}\times CEle{c}_{c,y}\times \left(1-{\alpha }_{c,d}\right),$$

10 $${\alpha }_{c,d}=\sum _{i}{\delta }_{d,j}\times {\mu }_{i,j},$$

where *α*_*c,d*_ is daily power consumption changes percentage on periods of the national lockdown in 2020, (which set to 0 in the regular year). For cities without complete daily power consumption data in 2020, the impact of the lockdown is estimated using adjacent city (in terms of change percentage *δ*_*d,j*_) that has actual daily power consumption data in 2020, adjusted by the industry proportion of the city *μ*_*i,j*_. The subscript *p* represents the province, *y* represents yearly, *i* represents the primary, secondary, and tertiary industries, and the subscript *j* corresponds to quarter, respectively.

#### Industry

For the industry sector, the daily emissions are calculated from the monthly industrial production and Carbon Monitor’s provincial NRT daily industry emissions. Monthly industrial production of 35 industrial products related to emissions are acquired from the National Bureau of Statistics of China (https://data.stats.gov.cn/) (Table 3) to construct the provincial weight factors of industrial CO_2_ emissions. We also divide the industrial sector in China subdivided into 10 subsectors in accordance with the Emission Database for Global Atmospheric Research (EDGAR) dataset^34,35^ classification standard and collect the provincial monthly data of 35 types of products relevant to emission (Table 3). Note that wood and wood products are not covered due to data availability, which only accounts for 0.11% of the total emission of the manufacturing industry based on EDGAR 2019. We conduct an assessment of the industrial subsectors for China in EDGAR 2019 and CEADs, and estimate that these 35 industrial products account for about 90% of the manufacturing industries and construction sector.

The monthly CO_2_ emissions estimated from the industrial production are then disaggregated into a daily scale following the Carbon Monitor’s provincial NRT daily industry emissions which is found to be correlated with daily power consumption^29^. We assume that the daily variation in a city’s industrial emissions follow the same pattern as its mother province for two reasons: 1. Daily and monthly industrial production at city-level are mostly unavailable. 2. Most high-consumption industrial plants in each province are located in selected cities based on our city selection criteria, which means that the relative changes in daily industrial activities in these cities can reasonably represent activities in corresponding provinces, and vice versa. Specifically, daily emissions from the industry sector is estimated as:

11 $$Emi{s}_{ind,c,d}=\left.\sum _{m}\left(Emi{s}_{ind,p,d}\times {R}_{i,m}\right)\times \left(\sum _{k}\frac{I{P}_{c,k}}{I{P}_{p,k}}\right)\times 1/k\right),$$

where *Emis*_*ind,c,d*_ is the industry emissions for city *c* on day *d*. *R*_*i,m*_ corresponds to the share of the industrial product category *i* in the total manufacturing industry emissions in month *m*. *IP* is the production of the industrial product. The subscript *p* stands for province (i.e., *Emis*_*ind,p,d*_ is the industry emissions in Carbon Monitor China for province *p*), and *k* is the number of relevant products in category *i*.

#### Ground transportation

Near-real-time daily emissions from ground transportation are estimated using Gaode (AMAP)(https://report.amap.com/index.do) live traffic data. Gaode congestion and delays indicators represent the ratio of the actual trip time to the trip time in the uncongested conditions. Congestion and delays indicators are proven to be a good proxy for daily on-road car flux^29^. For the 48 cities discussed in this paper, there are 44 cities with available Gaode daily congestion and delays data. We assume that the daily-scale distribution ratio of congestion and delays indicators in cities are the same as the most similar ones, such that the data of the remaining 4 cities are replaced by available data from the most similar city by using the city similarity matrix described in previous section.

As discussed by Liu *et al*.^29^, congestion and delays indicators with a value of 1 mean that the traffic is fluid or ‘normal’ and does not mean there are no vehicles and zero emissions. The lower threshold of emissions (when indicator = 1) is estimated using real-time traffic flow for all roads from December 26, 2021 to March 29, 2022 in Beijing. The daily traffic flow in Beijing is provided by Gaode, (traffic flow in this paper is defined as the mean number of passing vehicles on a road that were recorded by the camera within one hour). When the congestion and delays indicators are relatively low, with the increase of the congestion and delays indicators, the traffic flow gradually increases. The increase of the congestion and delays indicators reduces the speed of the vehicle at the same time, thus the traffic flow does not increase indefinitely but tends to be stable. To fit this flow model, we use a sigmoid function to represent the relationship between congestion and delays indicators X and flow Q,

12 $$Q=a+\frac{\beta {\left[100(X-1)\right]}^{\gamma }}{{\lambda }^{\gamma }+{\left[100(X-1)\right]}^{\gamma }},$$

where *Q* is Gaode traffic flow in Beijing, *X* is Gaode daily congestion and delays indicators and *a*, *β*, *γ*, *λ* are regression parameters.

**Fig. 4:**
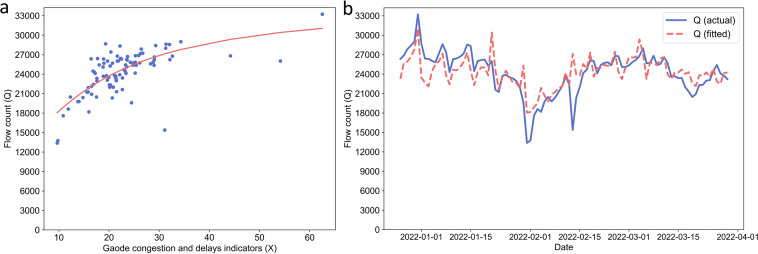
The relationship between Gaode congestion and delays indicators (*X*) with daily traffic flow (*Q*) for Beijing from December 26, 2021 to March 29, 2022. (**a**) The regression between *X* (x-axis) and *Q* (y-axis); (**b**) Comparison of reconstructed *Q* based on congestion and delay indicators (red) and the actual *Q* (blue).

We use the least squares method to fit the above model. Considering actual traffic flow, it is necessary to maintain traffic flow to be positive when the congestion and delays indicators equal 1. The fitting results are presented in Table 4. The flow model fitted in this paper is close to the real traffic flow in Beijing (Fig. 4). We assume daily flow for other cities follow a similar relationship to fitting models in Beijing (Eq. 12) and apply this model to the other 47 cities. This is an assumption as traffic characteristics vary across cities. However, due to data availability, we were only able to get Beijing’s traffic flow data at current stage. It is a reasonable approximation as the traffic condition of Beijing is known to be a good representation of the country’s average given its high diversity in road types (both modern and traditional roads and highways versus local roads, wider new paved roads versus narrower old roads). We are in the process of acquiring car flux data for more cities, In future updates, we will use city-specific regression models to improve the accuracy.

**Table 4:** Regression parameters of the sigmoid function of Eq. 12 that describes the relationship between traffic flow (Q) and congestion and delays indicators (X).

Regression Parameter	Value
*a*	11089.30
*β*	23460.82
*λ*	1.40
*γ*	17.93

We assume a linear relationship between daily CO2 emissions and daily traffic flow. The daily CO2 emissions from ground transportation are established based on annual CO2 emissions and daily traffic flow as the distribution coefficient. For a specific city *c*, the daily ground transportation emissions *Emis*_*tran,c,d*_ in day *d* are given by:

13 $$Emi{s}_{tran,c,d}={Q}_{c,d}\times \frac{Emi{s}_{tran,c,y}}{{\sum }_{d}{Q}_{c,d}}$$

where *Emis*_*tran,c,d*_ is the ground transportation emissions for city *c* in day *d*, *Emis*_*tran,c,y*_ is the ground transportation emissions for city *c* in year *y* and *Q*_*c,d*_ is traffic flow for city *c* in day *d*.

We assume a linear relationship between annual CO_2_ emissions of ground transportation and traffic flow. Then, the daily CO_2_ emission model of ground transportation is established. The daily emissions of 48 cities in China are calculated from January 1, 2020 to December 31, 2021. For a specific city *c*, daily ground transportation emissions *Emis*_*trans,c,d*_ in day *d* are given by:

14 $$Emi{s}_{tran,c,d}={Q}_{c,d}\times \frac{Emi{s}_{c,2020}}{{\sum }_{d=1}^{366}{Q}_{c,d}},$$

where *Emis*_*trans,c,d*_ is the ground transportation emissions for city *c* in day *d*, *Emis*_*trans,c,2020*_ is the ground transportation emissions for city *c* in 2020 and *Q*_*c,d*_ is traffic flow for city *c* in day *d*.

#### Residential: buildings

Daily direct emissions from the residential and commercial buildings are estimated by combining China Building Energy Model (CBEM)^36^ with the annual total building emissions calculated from EBTs following the China building energy consumption calculation method (CBECM)^37^. The daily emissions from the buildings are computed as:

15 $$Emi{s}_{bld,c,d}=\sum _{f}\left({E}_{bld,c,f}\times E{F}_{f}\times \frac{{\sum }_{bt}{r}_{bt,c}\times BE{C}_{bt,c,d}}{{\sum }_{bt}{r}_{bt,c}\times BE{C}_{bt,c,y}}\right),$$

where *E*_*bld,c,d*_ is the daily emissions from buildings in city *c*, *E*_*bld,c,f*_ is the fuel *f* consumed by all buildings in city *c*. *r*_*bt,c*_ is the share of building type *bt* in city *c*, *BEC*_*bt,c,d*_ and *BEC*_*bt,c,y*_ are the daily and yearly building energy consumption modeled for building type *bt* in city *c*, respectively. The annual building-related energy consumption is calculated from EBTs following CBECM^37^:

16 $${E}_{bld}={E}_{W}+{E}_{O}+{E}_{H}-{E}_{t}+{E}_{Y{B}_{heating}}-{E}_{{W}_{heating}}-{E}_{{O}_{heating}}-{E}_{{H}_{heating}},$$

where *E*_*W*_ is the energy consumption in wholesale and retail trades, hotels and catering services sector, *E*_*O*_ is the consumption in other sectors, *E*_*H*_ is the household (urban and rural) consumption. *E*_*t*_ is computed as the sum of 35% diesel and 95% gasoline consumption in *E*_*W*_ and *E*_*O*_ plus 100% diesel and 95% gasoline consumption in *E*_*H*_, *E*_*h*_ represents the heating consumption that is not covered in the EBTs, which is estimated by subtracting the heating component in *E*_*W*_, *E*_*O*_ and *E*_*H*_ from *E*_*YB_heating*_, which is the central heating supply data acquired and partitioned to cities from the China Statistical Yearbook.

All buildings are classified into 4 classes: 1. Residential buildings, 2. Office buildings, 3. Commercial buildings (retail and catering). 4. Public and other buildings. Each class is presented by a corresponding building model in the China Building Energy Model, and we simulated their daily building energy consumption (*BEC*) (except for electricity, which is covered in the power sector). The building model simulation is driven by daily air temperature collected at local weather stations for each city as air temperature is the dominant factor governing building energy use (e.g., heating demand) for all building classes.

We estimate the share of each building class in each city using building stock GIS data and POIs (point of interests). A POI table describes the function of each building and its geographic location, and provides classification for all the buildings, in which 9 classes are closely related to building energy consumption (Table 5). Since we only have four more general building models, the 9 classes are then grouped into 4 classes as listed in Table 5. We consider these 4 major classes can reasonably represent the daily patterns of building energy consumptions from a city. The building boundaries and point-based POIs are acquired from Amap’s open data platform (https://lbs.amap.com). The POIs of all buildings in a city are spatially matched to corresponding building boundaries using the Spatial Join function in ArcMap, and then associate the corresponding building surface area to each of the 4 building classes. By aggregating the surface areas for buildings that belong to each class, we calculate the share of different building types in terms of their building surface area for each city.

**Table 5:** Classification of urban buildings for each city.

POI code	Original Classification	Grouped Classification
50000	Restaurants	Malls and restaurants
60000	Shopping	Malls and restaurants
80000	Sports related services	Public and other buildings
100000	Hotels and lodging	Residential buildings
120000	Residential	Residential buildings
130000	Government and organizations	Public and other buildings
140000	Schools, research and education	Public and other buildings
160000	Financial and insurance	Office buildings
170000	Corporate and business	Office buildings

#### Residential: services

The residential sector also covers some non-building-related fuel consumption in wholesales, retail trade, catering and other small business, which we define as services. To estimate the daily activities for the services sub-sector, We distribute annual service-related activities into each day using city-level consumer goods retail sales plus the effects of weekends and major holidays. The consumer goods retail sales data is calculated from input-output (IO) tables which we constructed for each city. This approach assumes that a linear relationship exists between daily retail sales and daily energy consumption in the service sector. We first collected monthly statistics of total retail sales of consumer goods from city statistical yearbooks, then we used the monthly consumer price index to eliminate the effect of time on prices, which revised the monthly total retail sales of consumer goods. The monthly total retail sales of consumer goods are then allocated to each day following the moving holiday effect of China, which is presented in the form of holiday impact factors, including weekends and festivals^38,39^. China uses both the Gregorian calendar and the lunar calendar. For example, there are not only fixed holidays such as New Year’s Day, Women’s Day, Labor Day and National Day, but also moving holidays such as Spring Festival, Mid-Autumn Festival, Dragon Boat Festival and Qingming Festival. The total annual emissions for this sector are then disaggregated into a daily scale following:

17 $$Emi{s}_{service,c,d}=Emi{s}_{service,c,y}\times \frac{T{C}_{c,d}}{T{C}_{c,y}},$$

18 $$T{C}_{c,d}=T{C}_{c,m}\times MHIF,$$

where *Emis*_*service,c,d*_ is the daily service-related emissions for city *c* in day *d*, *Emis*_*service,y*_ is the yearly service-related emissions calculated based on the IO method, *TC*_*c,d*_, *TC*_*c,m*_ and *TC*_*c,y*_ are the daily, monthly and yearly total retail sales of consumer goods, respectively. *MHIF* is the moving holiday impact factor which is used to describe the effect of the holiday on the allocation of daily total retail sales of consumer goods. Specifically, MHIF is the ratio of daily resident consumption to monthly resident consumption, which is calcualted based on the decomposition analysis of the moving holiday components on resident consumption of China by Chen and Zhang^39^.

#### Aviation

Aviation CO_2_ emissions are calculated from the global flight reconstruction of Flightradar24 (https://www.flightradar24.com). Daily commercial flight data from 47 airports in China during the study period are acquired and attributed to each city (Shanghai and Beijing each has two airports, Binzhou, Suzhou and Dongguan do not have local commercial airports). Flightradar24 data is based on ADS-B signals emitted by aircraft and received by their network of ADS-B receptors. Each flight is characterized by its departure and arrival airports and an aircraft type. We calculate CO_2_ emissions from the flight data using the parameterization of Seymour *et al*.^40^, which takes into account a small deviation from the shortest flight route between the departure and arrival airports. We map the FlightRadar24 aircraft types onto the 133 aircraft types in Seymour *et al*.^40^. We have complemented their database by grouping together known similar aircraft with similar equipment and for aircraft not included in the study, the average coefficients have been computed (depending on their category, commercial planes or business jets). For flight data that do not contain any indication of aircraft type, the average coefficients of all aircraft in the study have been used. To avoid double counting and given the fact that most planes refuel before departure, the CO_2_ emissions are attributed to the city of departure.

### Limitations

The main purpose of this dataset is to improve the timeliness and temporal resolution of inventories for studying near-real-time fossil-fuel CO_2_ emissions and their responses to emergent events and short-term policies. CMCC does not include emissions related to land use, land use change, and forestry, therefore, some emissions caused by long-term urban expansion are not captured. CMCC is constructed based on daily activity data and models that can cover a majority rather than the entire daily emission-related activities due to data availability. Therefore, we acknowledge that a small portion of daily variations on city emissions are not reflected by this dataset. Another limitation is associated with the use of the city similarity matrix to find substitute cities to address the missing data issue, which may introduce additional uncertainties, but we consider our results to represent a meaningful first attempt to capture daily emissions for these cities.

### Code description

Python code for data generation and visualization is provided (link in the Code Availability section). The code disaggregates annual emissions calculated from energy statistics into daily resolution using daily activity data for each sector. For example, the “correct-save-flightradar24” function uses FlightRadar24 data to estimate daily emissions for each airport. Functions for other sectors follow similar process as detailed in previous sections. The “save-CM-cities” is used for saving data (all sectors) for individual city following a fixed format. The “merge-all-cities-in-folder” function merges all data into one excel sheet with each city as one sub-sheet. Lastly, users can plot the data for each city using the “plot-CM-cities” function.

## Data Records

CMCC includes city-level emission inventories from 01/01/2020 to 31/12/2021 for five main sectors: 1. Power generation, 2. Residential (buildings and services combined), 3. Industrial production, 4. Ground transportation, and 5. Aviation. Currently, CMCC covers 48 major cities in China. All data have gone through a validation process, in which we estimated the uncertainties and corrected errors.

The attributes of the final dataset are listed in Table 6, and the emission data are organized into spreadsheets, which contains 3655 rows of data for each of the 48 cities (5 sectors for each day). The supplementary sheet includes the complete results from uncertainty analysis and the complete city similarity matrix. The definitions for sectors are consistent with the Carbon Monitor national inventories. Table 3 lists the correspondence between CMCC sectors, CHRED sectors and the IPCC sectors. At the time of writing this article, this dataset has been updated to December 31, 2021, and future updates will also be released timely. The raw data can be found at figshare^41^.

**Table 6:** Data attributes.

Column	Description
City	Name of the city
Province	Name of the province where the city is located
Date	Date (YYYY-MM-DD) on which the emissions were estimated. Currently, the dataset provides emissions from 2020-01-01 to 2021-12-31
Sector	Sector for which the emissions were estimated, including power, industry, residential, ground transport, aviation
Value	Magnitude of daily emissions with a unit of *ktCO*_2_
Timestamp	Unix timestamp at 00:00:00 (GMT + 0000) on each day for scientific visualization

### Data examples

**Fig. 5:**
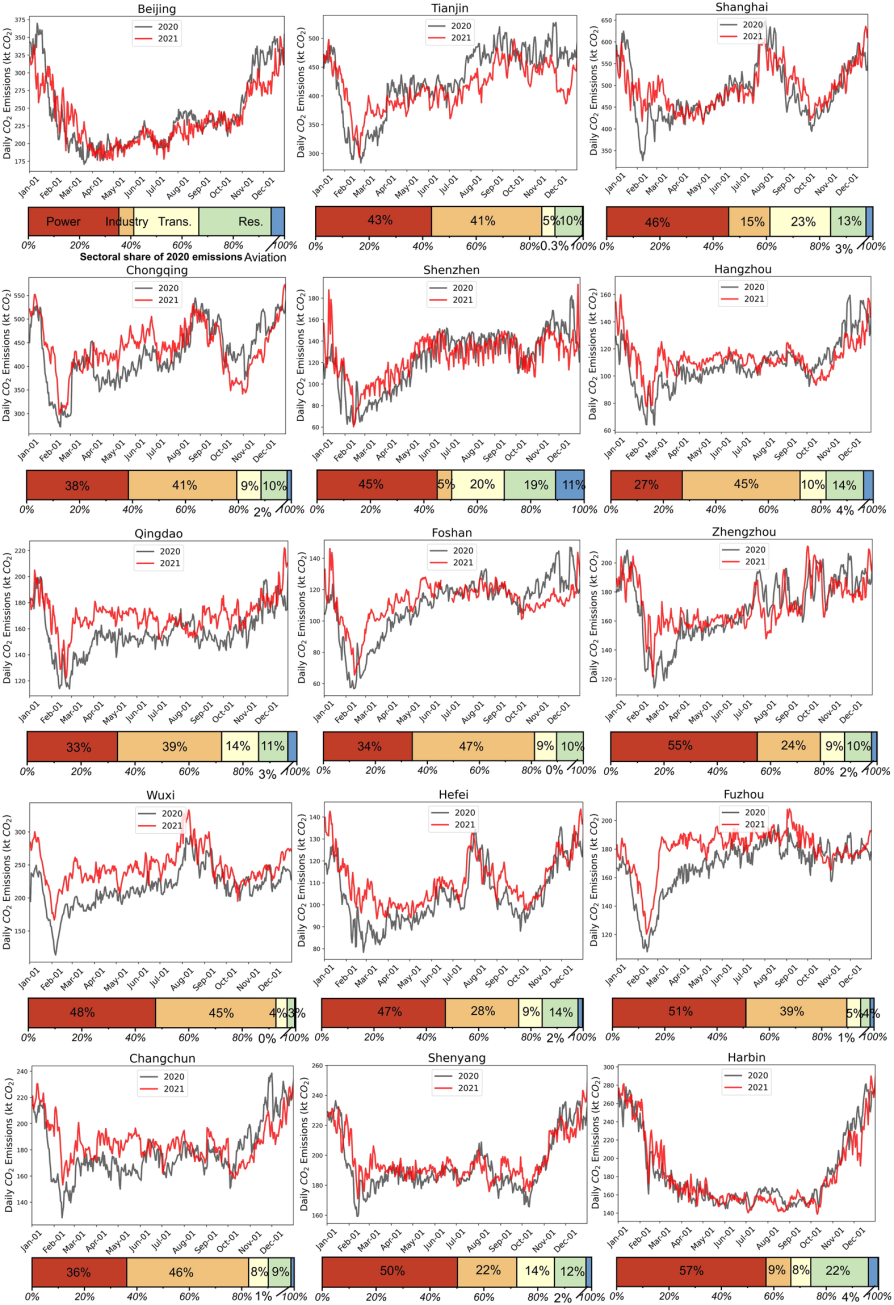
Daily total CO_2_ emissions for selected cities in the dataset. Gray lines represent emissions for 2020 and red lines represent emissions for 2021. Bars at the bottom illustrate the sectoral shares of 2020 annual CO_2_ emissions for each city.

Examples of daily CO_2_ emissions are presented here for selected cities. Figure 5 depicts the total daily emissions for year 2020 versus year 2021. By comparing the emissions in spring 2020 and spring 2021, we noted that for most cities, emissions rebound from the lower levels caused by COVID-19 pandemic. The bottom bar charts also illustrate the different sectoral shares of CO_2_ emissions for each city which reveal a city’s geographic and socio-economic characteristics.

**Fig. 6:**
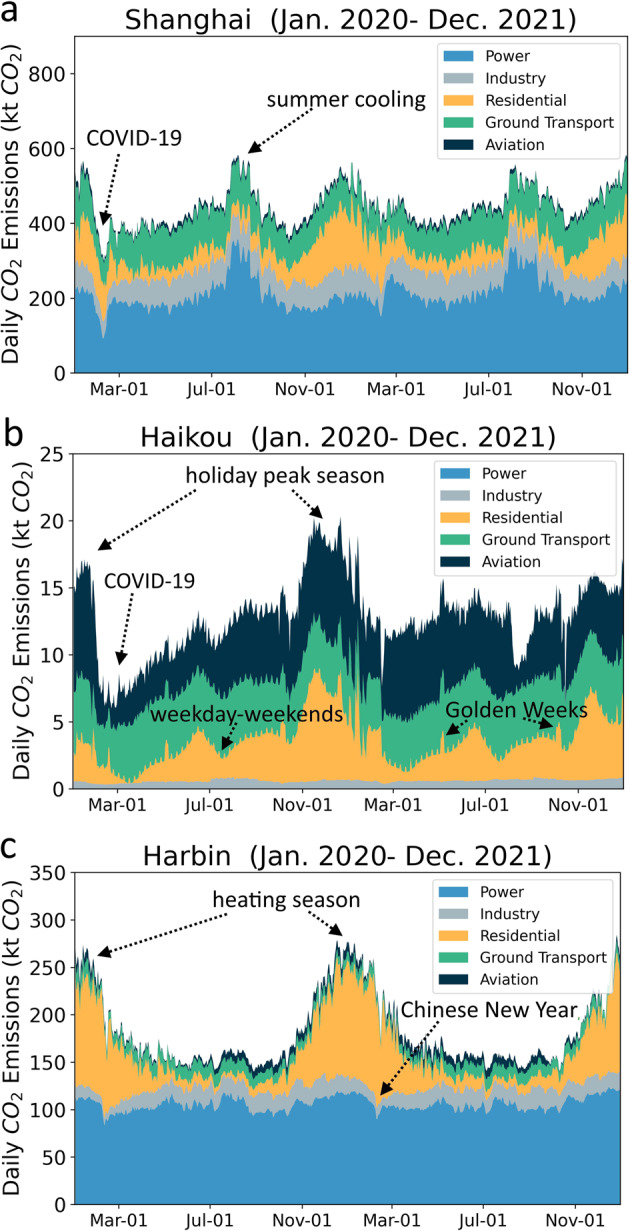
Daily by sector CO_2_ emissions for three cities in China. (**a**) Shanghai, a mega city highlights the impact of COVID-19 and summer cooling on emissions. (**b**) Haikou, the southernmost provincial capital city, exhibits a significant drop in aviation emissions during the winter holiday season in 2020. (**c**) Harbin, the northernmost provincial capital city, exhibits high emissions during the winter heating season.

Figure 6 shows the sectoral breakdown of daily CO_2_ emissions for 3 cities in different regions of China: Shanghai (Fig. 6a), a mega city on China’s central coast shows the drop in emissions due to COVID-19 lockdown in spring 2020. Power sector highlights a significant increase in emissions due to soaring summer cooling demand. As an example of the geographic influence on the emissions, we compare Haikou, a major city in the tropical zone (Fig. 6b) with Harbin, a major city in the north (Fig. 6c). We note that Haikou exhibits a large share of aviation emissions especially during the winter holiday season because it is a popular destination for winter vacations, but the pandemic caused a sudden drop in the aviation emissions starting from late January in 2020. Daily residential emissions reveal the emission changes during weekdays versus weekends and national holidays (e.g., the May 1st and October 1st Golden Weeks). Harbin, on the other hand, exhibits high residential emissions due to the high heating demand during its cold winter.

## Technical Validation

The quality of this dataset is assessed by comparing with existing datasets. We perform a comprehensive analysis of uncertainty for total and sectoral CO_2_ emissions at both annual and daily scales. Complete results for each stage of the uncertainty analysis is included in the supplementary data file.

### Validation CMCC against other datasets

Multiple datasets have been constructed to estimate annual CO_2_ emissions in China, including CEADs, MEIC, and CHRED. In this study, we validate our dataset CMCC by comparing its annual inventory with the above datasets. CEADs provides the total CO_2_ emissions at city level and sub-sector CO_2_ emission inventory at provincial level in China in 2019; we compare the total CO_2_ emissions for 40 ordinary cities and sub-sector CO_2_ emissions for 4 municipalities that are covered by both CEADs and CMCC in 2019. MEIC provides inventories of national and provincial CO_2_ emission data for 2017 and earlier; we compare only 4 municipality cities between MEIC and CMCC. CHRED provides a 5-year interval CO_2_ emission accounting inventory for Chinese cities from 2005 to 2020 and we compare CO_2_ emissions for the 48 cities in CHRED.

**Fig. 7:**
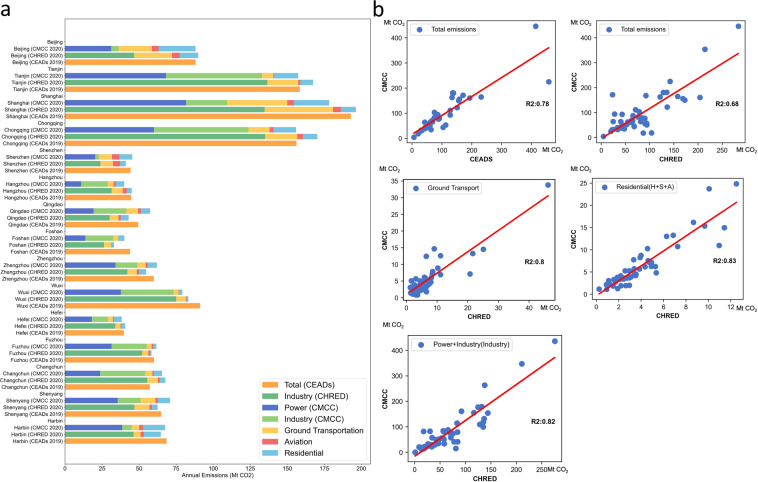
CMCC annual total emissions compared to CHRED and CEADs. (**a**) Annual total and sectoral comparisons between CMCC (2020), CHRED (2020) and CEADs (2019). (**b**) Subplots depict the coefficients of determination (R²) for the annual total and sectoral comparisons. Note that due to the different definitions of the residential sector, we take the sum of household, service and agriculture sectors as the residential sector for CHRED.

Figure 7a shows the comparison of annual total inventories, which indicates a good agreement among CMCC, CHRED and CEADs. Note that shipping related emissions (typically account for small percentage) are covered in CEADs for some cities but are not reflected in this comparison for CMCC and CHRED to keep the comparing sectors consistent. Figure 7b shows the comparison of annual sub-sector inventories. The coefficients of determination values (R²) between CHRED and CMCC are 0.82, 0.80 and 0.83 for the industrial sector, ground transport sector, and residential sector, respectively. The coefficient of determination values between CEADs and CMCC for 40 cities are 0.78 for the total emissions. As for the four municipality cities, the coefficient of determination values between CEADs and CMCC are 0.89 and 0.78 for the power sector and industry sector respectively. The coefficients of determination values between MEIC and CMCC for the four municipality cities are 0.90, 0.89, and 0.63 for the total emissions, power sector and industry sector respectively. The detail comparison results can be found in Table 7.

**Table 7:** Summary of city emission datasets (Δ is uncertainty, subscripts *y* and *d* represent yearly and daily, respectively) and comparison statistics including coefficient of determination (*R*^2^), sample size (*N*) when compared with CMCC.

Attribute	CMCC	CHRED	CEADs	MEIC
Spatial scale	48 Chinese cities	339 Chinese cities	China national,provincial, city, prefectural	China national, provincial
Temporal scale	2020–2021	2005–2020	1997–2019	2000–2017
Temporal resolution	Yearly/Daily	Yearly	Monthly	Yearly
Overall uncertainty (Δ_*y*_)	Δ_*y*_:2.54% to 30%	Δ_*y*_:7.22% to 33.63%^11^	Δ_*y*_: −15% to 30%^46^	Δ_*y*_: −15% to 15%^47,48^
Total emission comparison (With CMCC)	—	R^2^ = 0.68	R^2^ = 0.78	R^2^ = 0.90
		N = 48	N = 40	N = 4
Power comparison	Δ_*y*_:9.69% to 42.09%	—	R^2^ = 0.89	R^2^ = 0.89
	Δ_*d*_:7.81% to 31.6%		N = 4	N = 4
Industry comparison	Δ_*y*_:1.92% to 23.29%	R^2^ = 0.82	R^2^ = 0.78	R^2^ = 0.63
	Δ_*d*_:20.09% to 30.7%	N = 48	N = 4	N = 4
	Δ_*y*_:1.76% to 12.22%			
Residential comparison	Buildings:			
	Δ_*d*_:17.75% to 21.47%	R^2^ = 0.83	—	—
	Service:	N = 48		
	Δ_*d*_:7.29% to 14.12%			—
Ground transport comparison	Δ_*y*_:2.17% to 19.24%	R^2^ = 0.80		
	Δ_*d*_:18.95% to 26.92%	N = 48		
Aviation comparison	Δ_*d*_:-10.2% to 10.2%	—	—	—

### Uncertainty analysis

According to the IPCC guidelines, uncertainties may arise from each step when developing emissions accounting. In this study, we follow the 2006 IPCC Guidelines for National Greenhouse Gas Inventories^25^ to conduct an uncertainty analysis of the dataset to quantify the uncertainties of the total and sub-sectoral CO_2_ emissions at both annual and daily scales.

#### Error propagation for uncertainty quantification

**Fig. 8:**
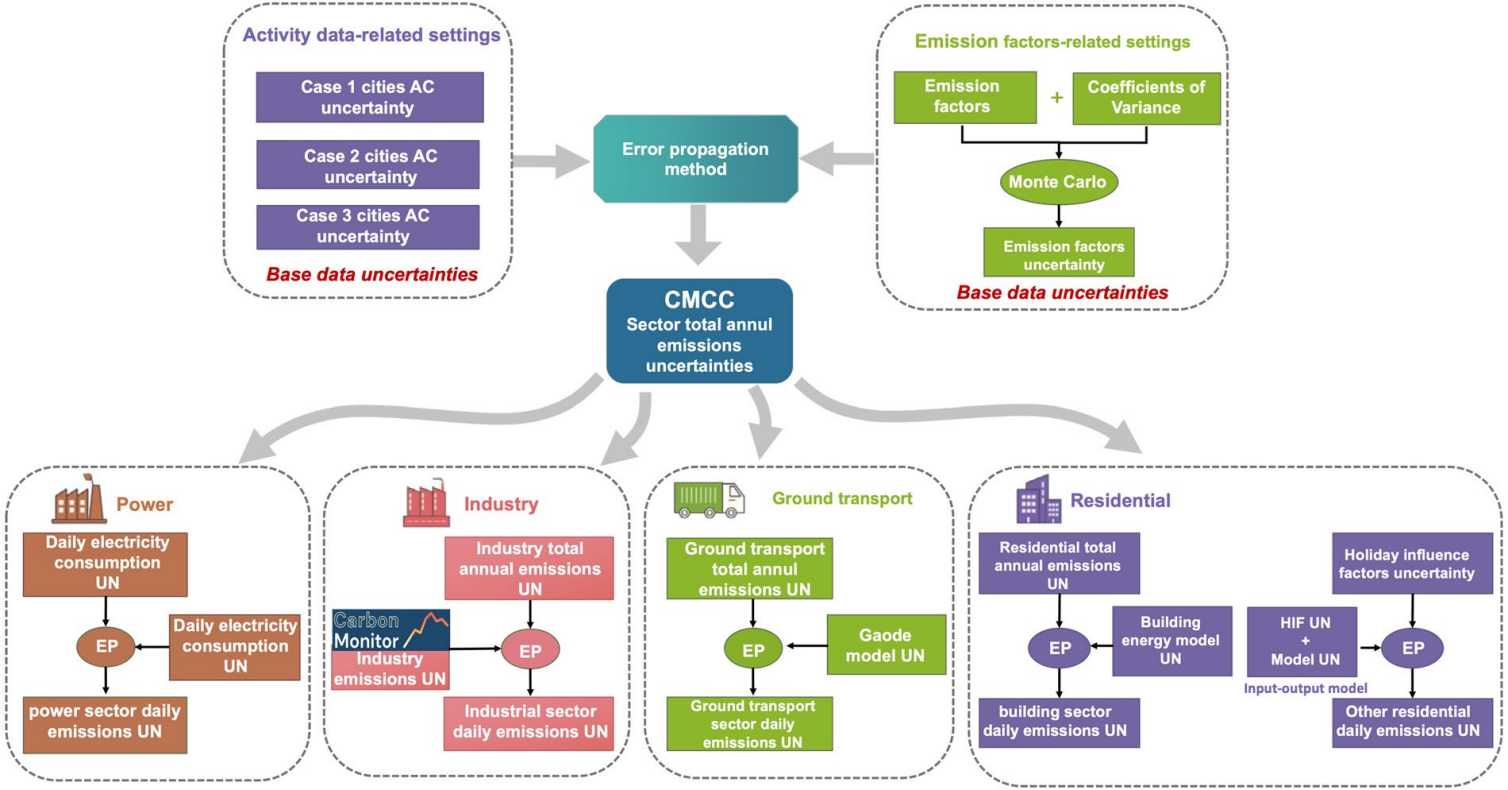
Diagram illustrates the uncertainty analysis workflow for each sector. Note that we quantified uncertainties not only for the annual bottom-up inventories but also for the daily activities and models. *EP* refers to error propagation method and *UN* refers to the uncertainties introduced by each step.

IPCC recommends two methods to quantify uncertainty: error propagation method and Monte Carlo simulation. The error propagation method follows a more rigorous uncertainty transfer process, allowing for a more accurate range of emissions uncertainty than the Monte Carlo simulation method. The key assumption in error propagation is that the propagation of emissions or uncertainties can be obtained from the uncertainty of activity data, emission factors and other estimated parameters through the error propagation formula. This process includes three steps: 1. Determination of the uncertainty of activity data, emission factors and other parameters used in different sectors; 2. Calculation of the corresponding emissions uncertainty of each industry according to the CO_2_ emission accounting process; 3. Combination of the uncertainty of each industry to obtain the uncertainty of total emissions. The calculation workflow is shown in Fig. 8. The error propagation process used in this work applies the following two equations. First, Eq. 19 is used for combining uncertainties of a single subsector:

19 $${U}_{i}=\sqrt{{U}_{AC}^{2}+{U}_{EF}^{2}},$$

where *U*_*AC*_ and *U*_*EF*_ are the uncertainty of activity data and emission factor data, respectively. After obtaining the uncertainty *U*_*i*_ of a single sector, it is applied with Eq. 20 to combine all subsector uncertainties to calculate the overall uncertainty:

20 $${U}_{total}=\frac{\sqrt{\sum {\left({U}_{i}\times {x}_{i}\right)}^{2}}}{\left|\sum {x}_{i}\right|},$$

where *x*_*i*_ is the CO_2_ emission of the corresponding subsector.

#### Uncertainty of annual fossil fuel consumption

The uncertainties (two sigma level) for annual fossil fuel consumption at city level are estimated following IPCC guidelines^25^, China Greenhouse Gas Inventory Study 2005 (CGGI 2005^42^), and city data quality expert advice. Based on the above-mentioned annual inventory construction workflow, the 48 cities of mainland China can be divided into three cases and further related to different levels of uncertainty of energy-related activity.

For case 1 cities, the statistical system is considered to be complete according to IPCC guidelines. The recommended uncertainties of main power production and heat production data is within 3%–5%, and we set it to 4%. The recommended uncertainties of the annual fossil fuel consumption for industrial combustion (energy intensive industries) is within 3%–5%, and we set it to 4%. The recommended uncertainties of commercial, institutional and residential combustion is within 5%–10%, and we set it to 5%. For other industries, according to the reference value provided by IPCC, is set to 10%. For case 2 cities, the statistical system is considered to be underdeveloped due to the lack of actual end-user consumption data (except the industrial sector). At the same time, the industry-wide output value for case 2 cities is estimated by the industrial sector gross value, which brings higher uncertainty than that of case 1 cities, and is set between 5% and 25%. For case 3 cities, there are fewest sources of data, and hence the highest data uncertainty. In line with IPCC, the uncertainty range of annual fossil fuel consumption for case 3 cities is within 20–40%.

It is noted that the input data of EPCT is subtracted from the corresponding industry in the emission accounting process, and the non-energy utilization data is subtracted from the end-user consumption data of the industrial industry. The uncertainty introduced in the above processes is so small compared to the uncertainty caused by missing data so that it is ignored.

Annual fossil fuel consumption for industrial processes (mainly cement) are derived from municipal government statistics, with reference to the calculation of CO_2_ emissions from China’s provincial cement production process^43^, with the uncertainty set to 5%.

#### Uncertainty of emission factors

For industry energy consumption-related emission factors, the distributions are assumed to be normally distributed if the coefficient of variance (CV) of the emission factor is less than 30% according to the IPCC guidelines. The 95% uncertainty range of the emission factors at 95% confidence interval are calculated with emission factors collected from IPCC and CEADs and their coefficients of variance^29,44^. The uncertainty of emission factors for industrial processes (cement) is mainly based on the range recommended by IPCC and CGGI 2005 (1%–2%), and is set to 1.5%. The uncertainties for annual fossil fuel consumption and emission factors are appended in our inventory.

#### Annual total and subsector uncertainties

After obtaining the uncertainty of annual fossil fuel consumption and the uncertainty of emission factors, the uncertainties of total CO_2_ emissions and sub-sectors of CO_2_ emissions for 48 cities in 2020 can be estimated using the error propagation method.

The uncertainty of annual total CO_2_ emissions for 48 cities ranges from 2.54% to 30.72%. Among 48 cities, the annual total emissions of 20 cities have uncertainties less than 10%, 21 cities between 10% - 20%, and 7 cities between 20% - 31%. City-specific uncertainty ranges for the annual inventories are listed in Table 8.

**Table 8:** Uncertainties for the annual CO_2_ emissions inventories.

City	Uncertainty				
	Total emissions	Transport	Residential	Power	Industry
Tianjin	4.31%	2.17%	2.14%	8.69%	5.12%
Shanghai	3.60%	2.50%	1.76%	7.94%	2.23%
Beijing	2.54%	3.23%	2.60%	6.26%	1.92%
Tangshan	5.50%	3.03%	3.15%	11.66%	6.52%
Chongqing	3.94%	2.28%	1.90%	9.94%	2.61%
Weifang	10.75%	16.37%	6.55%	24.10%	9.68%
Nanjig	8.69%	13.40%	10.87%	25.67%	9.39%
Foshan	8.38%	14.19%	10.46%	23.33%	5.02%
Shijiazhuang	8.82%	14.92%	6.00%	20.34%	12.79%
Zhengzhou	14.80%	16.90%	5.01%	26.57%	6.06%
Dalian	8.17%	15.07%	5.84%	27.01%	8.08%
Xuzhou	15.50%	13.40%	9.46%	27.23%	8.66%
Qingdao	9.21%	16.37%	5.88%	24.74%	7.48%
Yulin	13.21%	13.85%	5.49%	26.92%	14.79%
Xining	9.28%	18.16%	3.34%	25.60%	6.91%
Dongguan	9.94%	14.19%	7.61%	18.54%	15.15%
Taiyuan	7.49%	15.00%	6.54%	16.45%	9.13%
Ningbo	15.73%	13.91%	5.70%	27.24%	5.85%
Baotou	9.88%	10.91%	7.19%	26.79%	10.30%
Changsha	5.35%	12.81%	6.34%	25.80%	6.19%
Handan	10.10%	14.92%	7.19%	26.89%	11.67%
Harbin	23.79%	12.10%	12.19%	41.43%	10.66%
Wuhan	8.43%	13.30%	6.44%	28.01%	12.40%
Haikou	6.64%	16.89%	3.80%	6.87%	18.31%
Guiyang	7.89%	14.61%	10.32%	41.27%	9.20%
Linyi	19.15%	16.37%	6.48%	38.24%	11.81%
Shenzhen	13.18%	14.19%	3.34%	29.46%	13.45%
Nanning	11.03%	15.20%	12.22%	40.23%	11.69%
Suzhou	19.60%	13.40%	9.30%	41.43%	17.39%
Yinchuan	20.74%	19.24%	11.01%	39.12%	18.77%
Kunming	12.03%	15.98%	5.83%	34.73%	15.01%
Jinan	18.79%	16.37%	6.25%	41.42%	12.16%
Luliang	13.38%	15.00%	10.46%	41.06%	17.06%
Xi’an	20.77%	13.85%	9.54%	37.67%	9.73%
Huhhot	30.72%	10.91%	8.45%	41.44%	15.88%
Lanzhou	15.48%	16.36%	6.98%	37.83%	19.30%
Wuxi	17.94%	13.40%	9.26%	35.72%	12.91%
Urumqi	12.51%	15.71%	8.93%	34.35%	11.93%
Guangzhou	13.53%	14.19%	2.91%	41.39%	13.06%
Fuzhou	21.40%	12.35%	4.89%	41.43%	15.19%
Ordos	16.53%	10.91%	9.46%	40.82%	17.78%
Hefei	19.08%	16.77%	6.90%	41.43%	9.03%
Hangzhou	8.92%	13.91%	7.42%	27.80%	10.03%
Shenyang	21.24%	15.07%	3.79%	40.98%	23.29%
Changchun	17.69%	16.08%	7.48%	41.16%	20.48%
Nanchang	11.89%	17.13%	4.27%	41.18%	13.36%
Chengdu	5.01%	14.42%	5.89%	39.82%	6.63%
Binzhou	21.43%	16.37%	6.73%	39.95%	10.55%

**Fig. 9:**
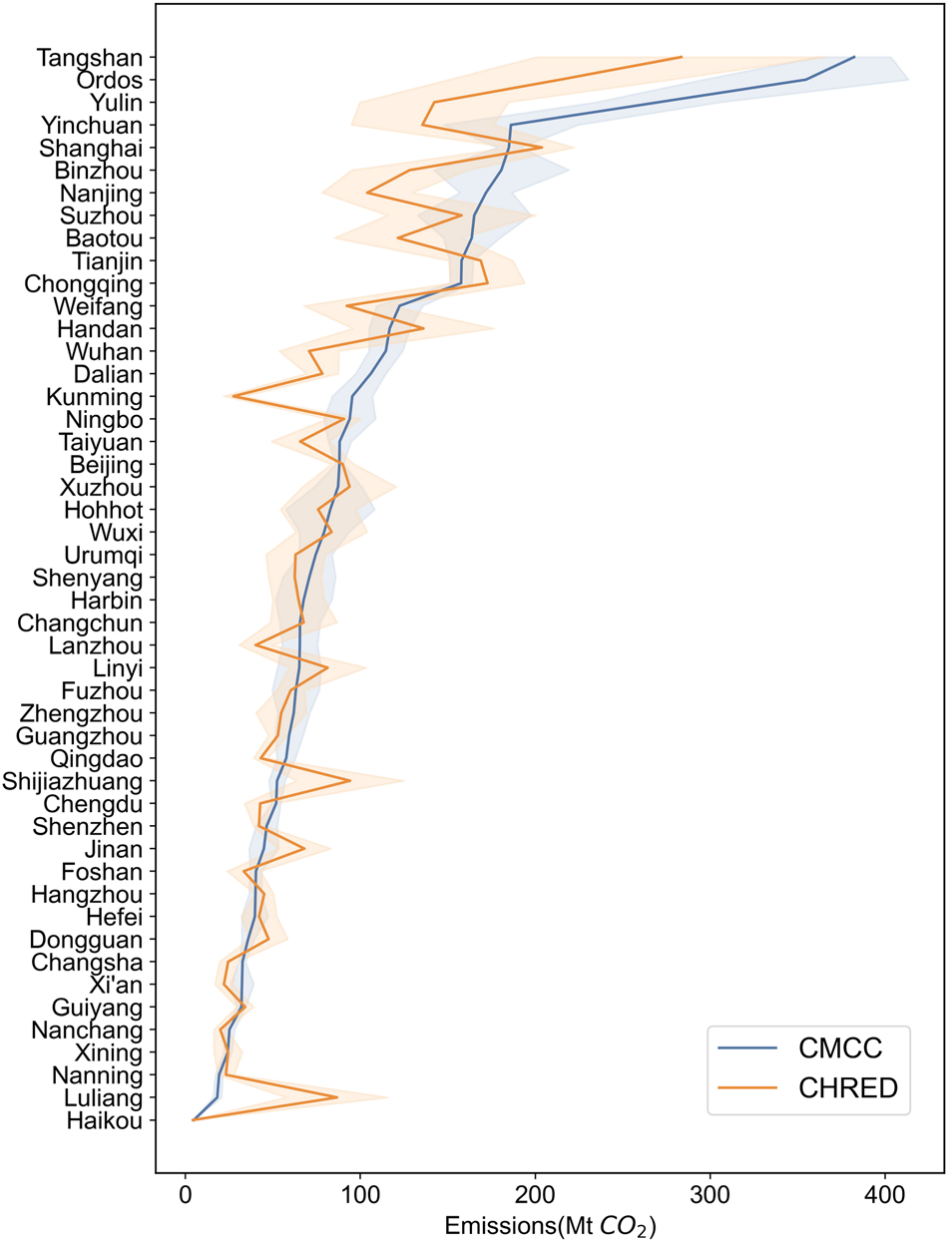
Overall emissions magnitude and uncertainties (shaded area) for all cities compared to uncertainty ranges in CHRED.

It is reasonable that the uncertainties tend to be lower in the case 1 cities, while the uncertainties are higher in the case 3 cities. The uncertainties of case 1 cities is generally lower than 6%. For example, Beijing’s total CO_2_ emissions has the lowest uncertainty among 48 cities, and the uncertainty of Tangshan’s total CO_2_ emissions is the highest among the case 1 cities, at 5.5%; The uncertainties of case 2 cities total CO_2_ emissions are between 5.35% and 15.73%, whereas the uncertainties of case 3 cities total CO_2_ emissions are between 5.01% and 30.72%. The last 5 cities in the emission uncertainty ranking all belong to case 3 cities, Hohhot has the highest total emissions uncertainty among 48 cities, at 30.72%. As shown in Fig. 9, the total CO_2_ emissions uncertainty of most cities in the CMCC is lower than those in the CHRED inventory, which is reasonable that our data adopts a top-down accounting method, and the sector’s coverage is more comprehensive.

#### Uncertainty of daily city emissions

In this section, we focus on the estimation of daily uncertainty from each sector, and the uncertainty of daily city emissions can be calculated by aggregating the uncertainty from each sector through the error propagation method. Note that adding daily uncertainties will increase the overall uncertainties on top of the annual uncertainties, which is not inconsistent because data are temporally auto-correlated^20^.

For the power sector, the uncertainty of daily-scale CO_2_ emissions has two sources: the first part of the uncertainty lies in the assumption that the CO_2_ emissions from daily power generation can be replaced and represented by the CO_2_ emissions from daily power consumption, and the uncertainty of this process is beyond the scope of this paper; the second part of the uncertainty is mainly from the uncertainty of daily power consumption data itself, which is difficult to assess without further investigation. Therefore, based on the completeness of the daily power consumption data, the uncertainty of this part is divided into two categories: For cities with complete daily power consumption data covering the whole accounting period (2020 to 2021 in this dataset), the uncertainty is from the lack of sampling and the aging of equipment, which is relatively small and neglected. For cities with incomplete daily power consumption data, we compare the real load curve data and simulated load curve and calculate the mean absolute error (MAE) to obtain the uncertainty of the allocation parameters, which is 7.39%.

For the industry sector, the uncertainty is mainly from monthly statistics. Deng *et al*.^45^ discuss the uncertainty of China’s industrial sector is 20% with a confidence interval of 68%. We apply this result to the error propagation formula to obtain the uncertainty of daily scale CO_2_ emissions in the industry sector.

For the transportation sector, the uncertainty of emissions from the ground transportation sector has two parts, the regression model and the daily-scale allocation of CO_2_ emissions by traffic flow. The uncertainty quantification of the daily-scale allocation of CO_2_ emissions requires real daily emissions from ground transportation, which is difficult to obtain and ignored in this study. Therefore, we focus on the uncertainty generated by the regression model. We used the 95% confidence interval of the regression model to estimate the uncertainty generated by the model. Applying the fitting results to the other 47 cities could introduce additional uncertainty, but this uncertainty is difficult to quantify due to the lack of similar accounting data in other cities in China. The uncertainty of the model is involved in error propagation. The daily emissions of aviation and shipping account for a low proportion of the emissions of the entire transportation sector. Therefore, the uncertainty of CO_2_ emissions in this part is not specifically discussed. Two methods are compared to calculate the uncertainty of the daily emissions of the aviation sector in Liu *et al*.^29^, and the uncertainty is cited in this study.

For the residential sector, the source of uncertainty in the service sector is mainly derived from the error in the input-output model and the uncertainty of the moving holiday impact factor, which are given by model contributor’s advice, and set to 5% and 5%, respectively. The overall uncertainties of daily emissions are then computed using the error propagation method (Tables 9, 10).

**Table 9:** Uncertainties for daily activities and models with sectoral breakdown.

Sectors		Daily emissions uncertainty ranges
Power		10% to 42%
Industry		20% to 31%
Ground transport		19% to 27%
Aviation		±10%
Residential	Buildings	18% to 21%
	Service	7% to 14%

**Table 10:** Overall uncertainty estimates for each city accounting for daily uncertainties and annual uncertainties.

City	Uncertainty				
	Ground transport	Buildings	Service	Power	Industry
Tianjin	18.95%	17.79%	7.39%	11.41%	20.64%
Shanghai	19.00%	17.75%	7.29%	10.85%	20.12%
Beijing	19.11%	17.85%	7.54%	9.69%	20.09%
Tangshan	19.07%	17.94%	7.74%	13.80%	21.04%
Zhengzhou	18.97%	17.76%	7.32%	12.39%	20.17%
Weifang	24.95%	18.84%	9.64%	25.21%	22.22%
Nanjing	23.11%	20.73%	12.96%	26.71%	22.10%
Foshan	23.58%	20.52%	12.63%	24.47%	20.62%
Shijiazhuang	24.02%	18.65%	9.27%	21.64%	23.74%
Linyi	25.30%	18.35%	8.67%	27.58%	20.90%
Dalian	24.12%	18.60%	9.17%	28.00%	21.57%
Xuzhou	23.11%	20.03%	11.81%	28.22%	21.79%
Qingdao	24.95%	18.61%	9.20%	25.82%	21.35%
Yulin	23.38%	18.49%	8.95%	27.91%	24.88%
Xining	26.16%	17.97%	7.82%	26.65%	21.16%
Dongguan	23.58%	19.23%	10.39%	19.96%	25.09%
Taiyuan	24.07%	18.83%	9.63%	18.03%	21.98%
Ningbo	23.41%	18.56%	9.08%	28.22%	20.84%
Baotou	21.76%	19.06%	10.08%	27.79%	22.50%
Lanzhou	22.78%	18.76%	9.50%	26.84%	20.94%
Handan	24.02%	19.07%	10.09%	27.89%	23.16%
Harbin	22.38%	21.45%	14.09%	42.08%	22.66%
Wuhan	23.05%	18.79%	9.56%	28.97%	23.53%
Haikou	25.30%	18.06%	8.03%	10.09%	27.11%
Guiyang	23.83%	20.45%	12.51%	41.92%	22.02%
Chongqing	24.95%	18.81%	9.59%	38.95%	23.23%
Shenzhen	23.58%	17.97%	7.82%	29.46%	24.10%
Nanning	24.20%	21.47%	14.12%	40.91%	23.17%
Suzhou	23.11%	19.96%	11.68%	42.08%	26.50%
Yinchuan	26.92%	20.81%	13.08%	39.81%	27.43%
Kunming	24.70%	18.60%	9.16%	35.50%	25.01%
Jinan	24.95%	18.73%	9.44%	42.07%	23.41%
Luliang	24.07%	20.52%	12.63%	41.72%	26.29%
Xi’an	23.38%	20.07%	11.87%	38.39%	22.24%
Huhhot	21.76%	19.57%	11.02%	42.09%	25.54%
Changsha	24.94%	18.99%	9.94%	38.54%	27.79%
Wuxi	23.11%	19.94%	11.65%	36.48%	23.81%
Urumqi	24.52%	19.79%	11.39%	35.13%	23.29%
Guangzhou	23.58%	17.90%	7.65%	42.04%	23.89%
Fuzhou	22.52%	18.32%	8.60%	42.08%	25.11%
Ordos	21.76%	20.03%	11.81%	41.48%	26.76%
Hefei	25.22%	18.96%	9.88%	42.08%	21.95%
Hangzhou	23.41%	19.15%	10.25%	27.8%	22.37%
Shenyang	24.12%	18.06%	8.02%	41.64%	30.70%
Changchun	24.76%	19.18%	10.29%	41.82%	28.63%
Nanchang	25.46%	18.17%	8.26%	41.83%	24.05%
Chengdu	23.71%	18.61%	9.20%	40.50%	21.07%
Binzhou	24.95%	18.90%	9.76%	40.63%	22.61%

## Usage Notes

The dataset (CMCC-48-cities-v0629.xlsx) is available from figshare^41^ 10.6084/m9.figshare.20264277.v2. Each city has more than 3000 lines of data, which will take a long time to load in Excel. We recommend loading the data with a script that can handle large datasets. Users should also note that the unit of emissions in this dataset is *ktCO*_2_.

## Supplementary information

Supplementary information

## Data Availability

Python code for producing, reading and plotting data in the dataset is provided at https://github.com/dh107/Carbon-Monitor-Cities/.
